# Fetuin-A levels are increased in the adipose tissue of diabetic obese humans but not in circulation

**DOI:** 10.1186/s12944-018-0919-x

**Published:** 2018-12-22

**Authors:** Abdelkrim Khadir, Sina Kavalakatt, Dhanya Madhu, Maha Hammad, Sriraman Devarajan, Jaakko Tuomilehto, Ali Tiss

**Affiliations:** 1 Research Division, Dasman Diabetes Institute, Al Kuwayt, Kuwait; 2 Functional Proteomics & Metabolomics Unit, Dasman Diabetes Institute, P.O. Box 1180, 15462 Dasman, Kuwait

**Keywords:** Fetuin-A, Insulin resistance, Obesity, Adipose tissue, Physical exercise

## Abstract

**Background:**

The hepatokine fetuin-A is linked to obesity and type 2 diabetes, but its presence and expression in adipose tissue remain unclear. In this study, we aimed to assess the circulating levels of fetuin-A and its expression in subcutaneous adipose tissue (SAT) from diabetic and non-diabetic obese subjects and their modulation by exercise.

**Methods:**

SAT and blood were obtained from adults obese (diabetic, *n*=118 and non-diabetic, *n*=166) before and after a 3-month exercise program (diabetic, *n*=40 and non-diabetic, *n*=36, respectively). Plasma fetuin-A was assayed using ELISA. The presence and expression of fetuin-A in SAT, peripheral blood mononuclear cells (PBMCs) and cell lines (3T3-L1, THP-1, HepG2, RAW 264.7) were analysed using confocal microscopy, immunoblotting and qRT-PCR.

**Results:**

Plasma fetuin-A level did not significantly differ between diabetic and non-diabetic obese subjects. However, when the non-diabetic group was divided into metabolically healthy and unhealthy phenotypes, significantly higher fetuin-A level was observed in the unhealthy sub-group. Circulating fetuin-A was mainly associated with glycaemic markers. In SAT, fetuin-A protein level was significantly higher in the diabetic obese subjects but its mRNA was not detected. Similarly, fetuin-A protein was detected in PBMCs, but its mRNA was not. In line with this, the use of various cell lines and culture media indicated that the presence of fetuin-A in SAT and PBMCs was due to its uptake from circulation rather than its endogenous expression. Finally, physical exercise decreased fetuin-A levels in both plasma and SAT in both groups.

**Conclusions:**

Fetuin-A levels increased in association with diabetes in SAT but not in circulation in the obese subjects. Moreover, physical exercise decreased fetuin-A level. Fetuin-A potentially acts as a hepatokine taken up by other tissues, such as adipose tissue.

**Electronic supplementary material:**

The online version of this article (10.1186/s12944-018-0919-x) contains supplementary material, which is available to authorized users.

## Background

Metabolic diseases such as obesity and diabetes are characterised by insulin resistance in peripheral tissues [1]. These tissues communicate with each other via secreted mediators, such as adipokines, hepatokines and myokines, to maintain metabolic homeostasis [2]. Among these mediators, fetuin-A is mainly secreted by the liver in adulthood and is one of the first hepatokines to be linked to metabolic diseases [3]. Indeed, fetuin-A is a natural inhibitor of insulin receptor tyrosine kinase [4]. Accordingly, knockout mice for fetuin-A have a greater insulin sensitivity and are resistant to weight gain when fed on a high-fat diet, suggesting the contribution of this protein in insulin resistance in rodents [5, 6]. Several reports have supported that increased fetuin-A levels are related to obesity and diabetes. Indeed, studies on humans have reported the association of higher fetuin-A levels with insulin resistance and metabolic syndrome and which could be a predictor of diabetes; this was recently comprehensively reviewed by focusing on the relationship between fetuin-A and obesity and its complications [7]. Interestingly, diabetic obese adolescents showed significantly higher serum fetuin-A levels than the non-diabetic obese individuals [8], whereas the morbidly obese individuals displayed decreased fetuin-A levels after weight loss upon bariatric surgery [9]. Nevertheless, the causal association between fetuin-A and diabetes was not supported by a large randomised Mendelian study in general population [10].

Adding to the controversy, fetuin-A was reported to be proinflammatory in diabetes and non-alcoholic fatty liver disease (NAFLD) but anti-inflammatory and protective against calcification in cardiac and renal diseases [11]. NAFLD is known to increase risk of diabetes, cardiovascular diseases (CVD) in particular atherosclerosis, and chronic kidney disease with implication of hepatokines including fetuin-A [6, 12, 13]. Indeed, several studies found an association of fetuin-A with fat accumulation in the liver of obese adults [6, 14, 15]. However, the link between fetuin-A and atherosclerosis is more controversial [12, 16, 17]. For instance, some studies have suggested an association of CVD with high Fetuin-A levels whereas others have report the opposite [18, 19]. Furthermore, in a prospective study with multi-ethnic population, no overall association between serum fetuin-A and CVD events was found but fetuin-A was associated with CVD risk in subjects with impaired fasting glucose or diabetes [20]. These conflicting results might be related to the different localizations of atherosclerosis and the pleiotropic effects of fetuin-A that are reflected by its dual function as inhibitor of vascular ectopic calcification [21] and its association with insulin resistance, T2D, metabolic syndrome and NAFLD [6, 22, 23].

The expression of fetuin-A in various tissues is controversial. Despite the fact that fetuin-A is mainly secreted by the liver, it has also been recently reported that it is secreted by adipocytes and expressed in adipose tissue [24, 25]. Similarly, another group reported that bone osteocytes, considered as the site of fetuin-A storage when it has been taken up from the circulation, express and secrete fetuin-A under the modulation of FGF23 [26]. However, other studies have shown that although normal adult rat kidneys do not express fetuin-A mRNA, the protein has been detected in proximal tubule epithelial cells by immunostaining, suggesting that fetuin-A is reabsorbed from plasma [27]. In contrast, a previous report of an association between fetuin-A and BMI has suggested that exercise and lifestyle interventions modulate fetuin-A levels. However, little research has focused on the effects of these interventions, and available data regarding these effects are contradictory probably because of inconsistency in the duration, intensity and type of exercise [28].

Taking into consideration these controversial reports on the levels of fetuin-A in relation with obesity, diabetes and their complications, we hypothesize that fetuin-A levels are further increased in diabetic obese in comparison with non-diabetic obese. Thus, in this present study, we sought to evaluate the circulating fetuin-A levels in adult obese subjects with and without diabetes, whether fetuin-A is present and expressed in adipose tissue, and how physical exercise might affect its levels in obese diabetic versus non-diabetic.

## Material and methods

### Study population

The study was conducted between June 2013 and June 2015 at the Dasman Diabetes Institute (DDI) and included two groups of obese human adults, diabetic (*n*=166) and non-diabetic (*n*=118). Written informed consents were obtained from all participants before their enrolment. The study was approved by our Institutional Review Board and performed in line with the Helsinki Declaration principles. Subjects were excluded in case they had performed any type of physical exercise during the last 6 months before their enrolment or those with a major illness or using medications influencing their body composition.

### Exercise protocol and measurement of anthropometric indicators

Subjects satisfying eligibility criteria were enrolled at our institute Fitness and Rehabilitation Centre in a supervised exercise protocol for 3 months, as we described previously [29]. In this protocol, subject underwent a physical assessment at baseline and after finishing the exercise period and various parameters related to the body composition and fitness capacity were measured as we reported before [29].

### Sampling of blood and tissue

Venous blood after at least 8-hours fasting and SAT biopsies were collected before and after accomplishment of the 3-month exercise protocol. Ficoll–Hypaque density method was used to prepare PBMCs. Vacutainer tubes were used to prepare plasma samples before being aliquoted and then kept at −80°C till use. SAT samples (around 0.2g) were collected from the periumbilical area by surgical biopsy following local anaesthesia. After removal, biopsied tissue sample were rinsed in PBS and appropriately stored till use.

### Analysis of blood markers

Lipid and glucose levels were measured with a Siemens Dimension Chemistry Analyzer (Diamond Diagnostics, Holliston,MA, USA). Haemoglobin A1c (HbA1c) level were assessed with the Variant™ device (Bio-Rad, Hercules, CA, USA). hsCRP and Insulin levels were measured with the hsCRP ELISA kit (Biovendor, Asheville, NC, USA) and Mercodia Insulin ELISA (Mercodia AB,Uppsala, Sweden), respectively. Fetuin-A levels in plasma were assayed using ELISA (Biovendor, Modrice, Czech Republic). All assays were performed in accordance with the manufacturers’ instructions.

### Immunofluorescence (IF) confocal microscopy

Sections of SAT which were formalin-fixed and paraffin-embedded were prepared for IF analysis as we previously described [29]. Anti-fetuin-A (Santa-Cruz, USA) and anti-adiponectin (BioVision) were used. Human liver biopsies, used as positive staining control, were obtained from consented cancer patients and provided by DDI Tissue Biobank. Tissue sections were incubated in the presence of Alexa Fluor® 488-conjugated anti-rabbit secondary antibody (Molecular Probes). Nuclear staining was performed using 0.05% DAPI. Sections analysis was performed with a Zeiss LSM 710 confocal laser-scanning microscope, and representative areas photographs of the adipose tissue were taken at a X40 microscope objective.

### Cell culture and treatments

All cell lines were purchased from ATCC and reagents from Gibco, unless otherwise mentioned. Mouse pre-adipocyte cells (3T3-L1) were cultured in DMEM with 10% of bovine calf serum (BCS). Human monocytic cells (THP-1) were grown in RPMI1640 medium supplemented with 10% of fetal bovine serum (FBS) and 0.05 mM β-mercaptoethanol. Human liver cancer cells (HepG2) were cultured in Eagle’s minimum essential medium with 10% FBS. Mouse macrophage cells (RAW 264.7) were cultured in DMEM supplemented with 10% FBS. All media were supplemented with 100 units/mL penicillin–streptomycin. To study the effect of sera on the levels of fetuin-A, cells were serum-starved for 1 day and then cultured overnight in BCS, FBS or without serum. To differentiate 3T3-L1 cells to mature adipocytes, briefly, preadipocytes were cultured in a DMEM with 10% BCS in the presence of 100 units/mL of penicillin/streptomycin in a 5% CO_2_ incubator at 37°C. Following their confluence (day 1), cells were initiated for differentiation to adipocyte using the induction medium (DMEM and 0.5 mmol/L isobutylmethylxanthine, 10 mmol/L insulin and 0.25 mmol/L dexamethasone) supplemented with 10% FBS or BCS. After 2 of induction (day 3), the maintenance medium (DMEM with insulin) supplemented with 10% FBS or BCS was changed, with this change being then repeated every two days. Cells were then prepared for Oil Red O staining to confirm the differentiation to adipocyte.

### Quantitative real-time (qRT)-PCR

Total RNA was extracted from PBMCs and SAT with an AllPrep RNA/Protein Kit RNeasy Lipid Tissue Mini Kit (Qiagen, Valencia, CA, USA), respectively. TRIZOL method was used for RNA extraction from cell lines. cDNA synthesis from total RNA samples was performed with cDNA Reverse Transcription Kit (Applied Biosystems, Foster City,CA, USA). qRT-PCR was performed using a Rotor Gene Q-100 platform (Qiagen Valencia, CA, USA) and SYBR Green normalised with GAPDH. Differences in gene expression between groups were assessed using the ΔΔCT method, and GAPDH was used as an internal control for normalisation. Used primers for validation are summarised in Additional file 1: Table S1.

### Statistical analysis

SPSS software (v25.0; SPSS Inc., Chicago, IL, USA) was used to perform statistical analyses. All descriptive statistics for variables in this study are reported as mean± standard deviation. Data distribution were assessed using normality tests. Accordingly, for variables with a normal distribution a parametric t-test was used to assess the difference significance between the groups before the exercise, and the non-parametric Mann–Whitney t-test was used for analysis of the skewed variables. A paired t-test was used to assess the difference significance within each of the non-diabetic and diabetic groups separately at baseline and after exercise intervention. To evaluate the separate and the combined effects of groups and exercise intervention, we used two-way repeated measures ANOVA test. Effects of sample size and homogeneity on ANOVA results were assessed using the partial eta-squared, Levene’s test of equality and Box’s M test. Spearman's correlation coefficient was used to assess correlations between variables. For all analyses, statistically significant differences were considered for *p* < 0.05.

## Results

### Plasma fetuin-A levels are not affected by diabetes in obese but modulated by physical exercise

In this study, 166 non-diabetic (67 males and 99 females) and 118 diabetic (64 males and 54 females) subjects were enrolled. Characteristics at baseline and biochemical measurements of the studied population are summarised in Table 1. Significant differences between the groups were observed because diabetic subjects were older and had significantly higher waist circumference (WC), BMI, percent of body fat (PBF), WBC and systolic blood pressure (*p* < 0.05) but lower VO_2,max_ and resting HR (*p* < 0.001 and *p* = 0.006, respectively). The diabetic group had a higher level of TG (*p* < 0.001) and a lower level of HDL (*p* = 0.009). As expected for glycaemic index markers, significantly higher FBG and HbA1c (*p* < 0.001) were observed in diabetic subjects. Finally, levels of circulating fetuin-A and hsCRP were higher in diabetic subjects, although this did not show statistical significance.

**Table 1:** Characteristics of the study population at baseline

	Obese diabetic	Obese non-diabetic	*P* value
*Anthropometric and physical characteristics*			
Gender (Male/Female)	118(64/54)	166 (67/99)	**0.020**
Age (years)	52±9.4	40±11.6	**<0.001**
BMI (kg/m^2^)	31.43 ±4.50	29.19 ±5.83	**<0.001**
PBF (%)	36.60 ±8.08	34.42 ±6.70	**0.015**
Waist (cm)	105.19 ±11.36	96.80 ±14.51	**<0.001**
Hip (cm)	110.97 ±11.82	108.50 ±15.63	0.117
WBC10	7.34 ±1.93	6.57 ±1.79	**<0.001**
SBP (mmHg)	121.83 ±12.13	116.87 ±12.18	**0.001**
DBP (mmHg)	77.28 ±7.47	76.15 ±8.11	0.210
HR (beats/min)	82.52 ±13.04	78.55 ±11.17	**0.006**
V_O2, Max_ (ml/kg/min)	15.93 ±4.40	19.25 ±4.97	**<0.001**
*Medication*			
*Anti-diabetic*			
Metformin	72 %	1%	-
Insulin	36%	-	-
Sulfonylurea	20%	-	-
DPP4 inhibitors	17%	-	-
GLP1 analogues	3%	-	-
*Anti-hypertensive*	57%	10%	-
*Lipid lowering*	69%	8%	-
*Metabolic markers*			
Cholesterol (mmol/l)	4.98 ±1.29	5.07 ±0.92	0.489
HDL (mmol/l)	1.17 ±0.43	1.29 ±0.40	**0.009**
LDL (mmol/l)	3.07 ±1.24	3.21 ±0.85	0.205
TG (mmol/l)	1.72 ±1.07	1.25 ±1.25	**<0.001**
FBG (mmol/l)	9.04 ±3.64	5.30 ±0.72	**<0.001**
HbA1c (%)	7.33 ±1.91	5.89 ±0.52	**<0.001**
Insulin (ng/ml)	3.90 ±1.88	3.42 ±1.99	0.072
C-pep (ng/ml)	3.98 ±5.26	5.12 ±6.12	0.135
hsCRP (*μ*g/ml)	5.67 ±4.31	4.63 ±4.36	0.109
Fetuin-A (mg/ml)	1.27 ±0.33	1.15 ±0.34	0.070

Data are presented as mean ± SD. *PBF* Percent body fat, *BMI* Body mass index, *SBP* Systolic blood pressure, *DBP* Diastolic blood pressure, *LDL* Low density lipoprotein, *TG* Triglycerides, *HDL* High density lipoprotein, *C-pep* C-peptide, *hsCRP* High-sensitive C-Reactive Protein. Nonparametric Mann-Whitney test was used to determine significance of difference in means between obese diabetic and obese non-diabetic groups

Bold text: *p*-value <0.05

As displayed in Table 2 and Additional file 2: Figure S1, Spearman’s rank test analysis including all subjects showed positive correlations of fetuin-A with glycaemic markers FBG and HbA1c (*p* < 0.01 and *p* < 0.05, respectively) as well as with TG (*p* < 0.05), whereas fetuin-A was negatively correlated with HDL (*p* < 0.05). Furthermore, stepwise multivariate linear regression analysis was performed with fetuin-A as a dependent variable; for this, two models were applied, which showed that FBG, HbA1c and HDL were independently associated with circulating fetuin-A levels (Table 3).

**Table 2:** Spearman correlation of circulating fetuin-A from all subjects with population characteristics

Markers	*r*
Age (years)	-0.019
BMI (kg/m^2^)	-0.058
PBF (%)	-0.070
Waist (cm)	0.036
Hip (cm)	-0.054
WBC10	0.080
SBP (mmHg)	-0.028
DBP (mmHg)	0.128
HR (beats/min)	0.096
V_O2, Max_ (ml/kg/min)	-0.025
Cholesterol (mmol/l)	-0.011
HDL (mmol/l)	**-0.131** ^*^
LDL (mmol/l)	-0.012
TG (mmol/l)	**0.126** ^*^
FBG (mmol/l)	**0.176** ^**^
HbA1c (%)	**0.151** ^*^
Insulin (ng/ml)	0.042
C-pep (ng/ml)	0.079
hsCRP (*μ*g/ml)	0.024

* *p* < 0.05; ** *p* < 0.01

Bold text: *p*-value <0.05

**Table 3:** Multiple stepwise linear regression analysis for Fetuin-A predictors^a^

	β	*p* value
Model 1		
HDL	-0.115	0.021
Hb1Ac	0.029	0.047
Model 2		
FBG	0.211	0.009

Model 1: The following variables were included; age, gender, BMI, cholesterol, TG, HDL, LDL, FBG and HbA1c.

Model 2: The following variables were included; age, gender, weight, BMI, PBF, WC, HIP, SBP, DBP, V_O2,Max_, cholesterol, TG, HDL, LDL, FBG and HBA1C

^a^All subjects were included in this analysis

Further analyses of baseline characteristics in the diabetic group were performed by clustering subjects into two subgroups as those with good glycaemic control and those with poor glycaemic control based on HbA1c levels, as detailed in Additional file 1: Table S1. The group with poor metabolic control displayed clear and significant differences in glycaemia, adiposity markers, blood pressure and hsCRP but not in fetuin-A (*p* = 0.065) compared with the group with good metabolic control (Additional file 1: Table S1). Non-diabetic persons were divided into two groups as metabolically unhealthy obese (MUO) and metabolically healthy obese (MHO) phenotypes, as detailed in Additional file 3: Table S2. Interestingly, significantly higher circulating fetuin-A levels were observed in the MUO group than in the MHO group, highlighting the relationship between fetuin-A and components of metabolic syndrome (Additional file 3: Table S2).

As a next step, to assess the effect of 3 months of moderate physical exercise, a pairwise comparison of fetuin-A levels and other physical, clinical and metabolic parameters was conducted on a subset of subjects (*n* = 50 for each group). In both diabetic (Table 4) and non-diabetic (Table 5) groups, exercise significantly decreased fetuin-A levels in the plasma. Moreover, the effect of exercise on other markers was more prominent in the non-diabetic group, in which adiposity markers (BMI, WC and PBF) and insulin were statistically significantly decreased, along with ameliorated cardiorespiratory markers (HR and VO_2,max_). In the diabetic group, however, only HbA1c and TC significantly decreased after physical exercise intervention. To further investigate the combined effect of diabetes aside with exercise intervention, two-way ANOVA with repeated measures analysis was conducted. As shown in Additional file 4: Table S3, the findings on separate effects of exercise and diabetes agreed with the results observed when using paired t-test, with regards to adiposity, glycaemic and lipid markers. For most of these markers, statistically significant differences were still obtained in the combined effect of both diabetes and exercise; however, fetuin-A levels were not significantly affected (*p* = 0.302).

**Table 4:** Physical, clinical and biochemical characteristics of diabetic subjects before and after exercise

	Diabetic before	Diabetic after	*P* value
	(*n* = 40)		
*Anthropometric and physical characteristics*			
BMI (kg/m^2^)	32.06 ±3.99	31.75 ±3.62	0.153
PBF (%)	35.13 ±5.60	34.49 ±5.58	0.060
Waist (cm)	107.26 ±9.60	105.47 ±10.02	0.065
Hip (cm)	110.96 ±9.51	111.01 ±9.12	0.939
WBC10	7.70 ±1.86	7.50 ±1.89	0.500
SBP (mmHg)	118.75 ±15.00	121.43 ±8.23	0.463
DBP (mmHg)	75.62 ±6.30	75.75 ±5.38	0.955
HR (beats/min)	83.00 ±13.27	79.56 ±12.23	0.261
V_O2, Max_ (ml/kg/min)	18.37 ±3.96	19.08 ±5.45	0.597
*Metabolic markers*			
Cholesterol (mmol/l)	4.84 ±1.27	4.33 ±0.91	**0.011**
HDL (mmol/l)	1.11 ±0.42	1.06 ±0.32	0.194
LDL (mmol/l)	2.97 ±1.11	2.65 ±0.91	0.111
TG (mmol/l)	1.66 ±1.01	1.60 ±0.73	0.764
FBG (mmol/l)	8.09 ±2.96	8.17 ±2.98	0.851
HbA1c (%)	7.75 ±1.96	7.10 ±1.35	**0.001**
Insulin (ng/ml)	3.77 ±1.74	3.52 ±2.13	0.449
C-pep (ng/ml)	3.53 ±5.02	3.37 ±3.36	0.717
hsCRP (*μ*g/ml)	3.53 ±2.19	4.37 ±4.39	0.520
Fetuin-A (mg/ml)	1.21 ±0.33	1.08 ±0.44	**0.002**

Data are presented as mean ± SD. *PBF* Percent body fat, *BMI* Body mass index, *SBP* Systolic blood pressure, *DBP* Diastolic blood pressure, *TG* Triglycerides, *HDL* High density lipoprotein, *LDL* Low density lipoprotein, *C-pep* C-peptide, *hsCRP* High-sensitive C-Reactive Protein. Paired t-test was used to compare differences in diabetic at baseline and after physical exercise

Bold text: *p*-value <0.05

**Table 5:** Physical, clinical and biochemical characteristics of Non-diabetic subjects before and after exercise

	Non-Diabetic before	Non-Diabetic after	*P* value
	(*n*=36)		
*Anthropometric and physical characteristics*			
BMI (kg/m^2^)	29.00 ±4.99	28.34 ±4.58	**0.004**
PBF (%)	33.98 ±5.99	33.04 ±6.32	**0.004**
Waist (cm)	95.03 ±13.16	91.16 ±12.50	**<0.001**
Hip (cm)	108.74 ±15.53	105.38 ±9.28	0.128
WBC10	6.05 ±1.66	5.98 ±1.60	0.691
SBP (mmHg)	114.52 ±10.27	112.68 ±7.58	0.266
DBP (mmHg)	74.19 ±6.72	74.03 ±5.54	0.914
HR (beats/min)	82.48 ±9.13	77.30 ±12.56	**0.006**
V_O2, Max_ (ml/kg/min)	19.20 ±3.85	22.22 ±5.09	**<0.001**
*Metabolic markers*			
Cholesterol (mmol/l)	5.24 ±0.91	5.18 ±0.98	0.587
HDL (mmol/l)	1.44 ±0.52	1.40 ±0.43	0.594
LDL (mmol/l)	3.28 ±0.90	3.23 ±0.90	0.672
TG (mmol/l)	1.01 ±0.51	1.30 ±1.11	0.064
FBG (mmol/l)	5.21 ±0.57	5.49 ±0.98	0.067
HbA1c (%)	5.87 ±1.10	5.70 ±0.45	0.182
Insulin (ng/ml)	3.92 ±2.11	3.05 ±1.36	**0.009**
C-pep (ng/ml)	6.76 ±8.49	5.80 ±5.94	0.185
hsCRP (*μ*g/ml)	4.68 ±6.60	3.21 ±1.95	0.500
Fetuin-A (mg/ml)	1.15 ±0.31	1.07 ±0.45	**0.020**

Data are presented as mean ± SD. *PBF* Percent body fat, *BMI* Body mass index, *SBP* Systolic blood pressure, *DBP* Diastolic blood pressure, *HDL* High density lipoprotein, *LDL* Low density lipoprotein, *TG* Triglycerides, *C-pep* C-peptide, *hsCRP* High-sensitive C-Reactive Protein. Paired t-test was used to compare differences in Non-diabetic before and after physical exercise

Bold text: *p*-value <0.05

### Fetuin-A levels are increased in diabetic obese SAT and decreased by physical exercise

**Fig. 1:**
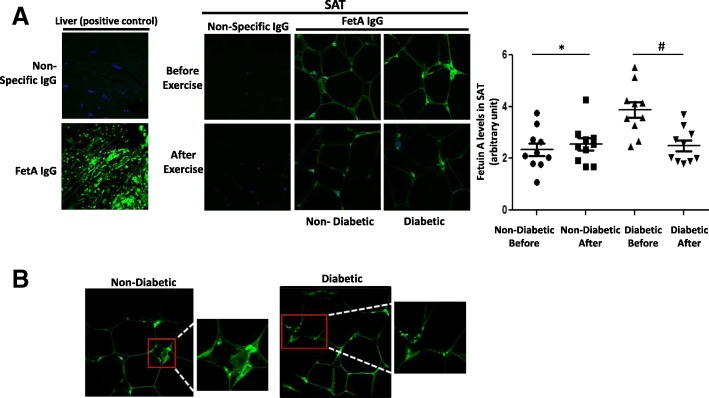
Immunofluorescent analysis of fetuin-A level and its modulation by exercise in subcutaneous adipose tissue (SAT) of obese subjects with and without diabetes. **a** Representative confocal immunofluorescence images illustrating the presence of fetuin-A in SAT from obese subjects without and with diabetes (*n* = 10 for each group). Densitometry quantification of SAT staining was performed as mentioned in the Materials and Methods section. The p value was determined using Mann–Whitney test for comparisons between the groups and using a paired *t*-test for intragroup comparisons before and after exercise. * *p* < 0.05 between diabetes and non-diabetes groups, # *p* < 0.05 between before and after exercise. **b** Confocal microscopy showing fetuin-A localising in vesicular-like structures in the thin rim of cytoplasm

As the difference in circulating fetuin-A levels was not statistically significant between the diabetic and non-diabetic obese, we investigated potential difference of fetuin-A levels in the SAT of those two subject groups. Furthermore, we assessed the potential effect of exercise on its levels. Confocal immunofluorescence revealed more intense fetuin-A staining in diabetic obese than in non-diabetic obese SAT, but much less intense staining than in human liver, which was used as a positive control (Fig. 1a). Fetuin-A staining was distributed throughout the thin rim of adipocyte cytoplasm, with intense positive staining in the stromal vascular zones. Moreover, a consistent punctate pattern of staining was observed, suggestive of fetuin-A localisation in vesicle-like structures (Fig. 1b). In contrast, a clear decrease in fetuin-A staining was observed after exercise in both diabetic and non-diabetic obese groups (Fig. 1a), in agreement with our results obtained from an analysis of blood (Tables 4 and 5). For further control, adiponectin staining levels were assessed in SAT from the same subjects, and interestingly, the levels showed the opposite pattern to fetuin-A levels; thus, diabetic subjects displayed lower adiponectin levels in their SAT than non-diabetic subjects (Additional file 5: Figure S2). In order to assess fetuin-A expression levels in SAT, RNA and RT-PCR analyses were performed; however, and in contrast with fetuin-A protein, fetuin-A transcript could not be detected in SAT samples from these subjects, whereas an abundance of adiponectin transcripts was detected (data not shown).

### Fetuin-A is taken up but not expressed by PBMCs

**Fig. 2:**
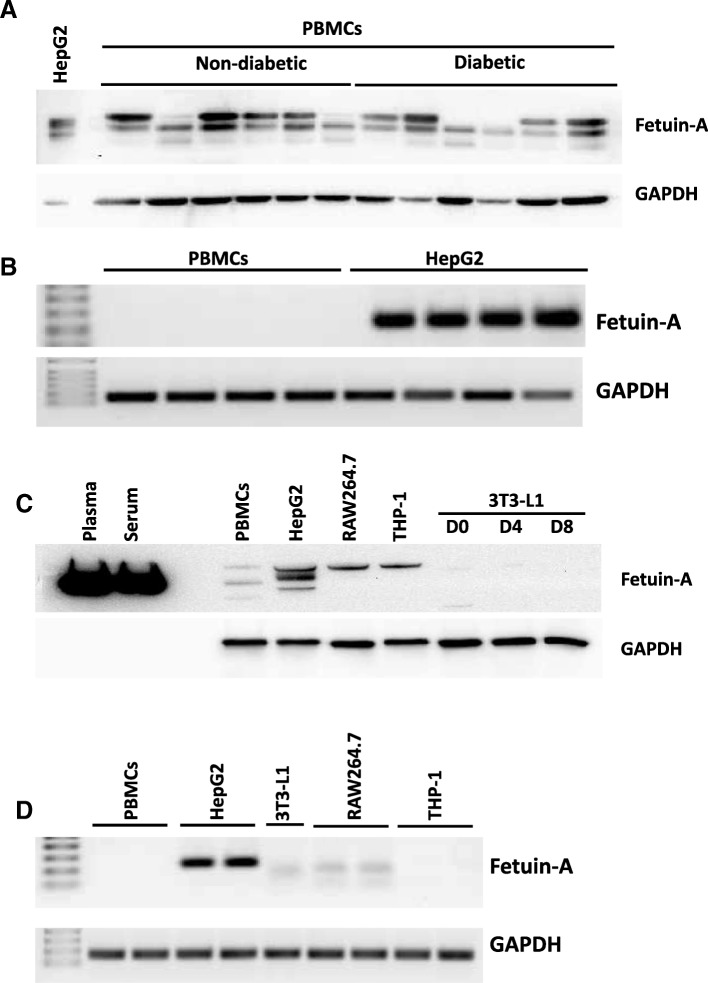
Fetuin-A levels in PBMCs from obese subjects with and without diabetes. **a** Total proteins were extracted from peripheral blood mononuclear cells (PBMCs) from obese subjects with and without diabetes (*n* = 12 each) and subjected to Western blotting. The blots shown here are representative of independent experiments with consistent results. Proteins extracted from HepG2 cells were used as a positive control and loaded on the gel at a level 10 times lower than that for PBMCs. Data are presented as fold change in non-diabetic obese subjects compared with diabetic obese subjects. **b** Total RNA was isolated from PBMCs and positive control HepG2 cells and subjected to qRT-PCR, as detailed in the Materials and Methods section. **c** Total proteins were extracted from cell lines and PBMCs and then subjected to Western blotting for fetuin-A. Human serum and plasma were used as a positive control. The blots shown here are representative of three independent experiments with consistent results. **d** Total RNA was isolated from cells and subjected to qRT-PCR as detailed in the Materials and Methods section

As mentioned above, high fetuin-A staining was present in SAT zones that seemed to be infiltrated by blood cells, such as macrophages, which were previously reported to take up fetuin-A from the blood [30]. Thus, using Western blotting, a protein analysis of PBMCs from both subject groups was performed; our results revealed the presence of fetuin-A protein in PBMCs (Fig. 2a). Notably, fetuin-A levels varied between individuals but without any significant difference in PBMCs between the non-diabetic and diabetic groups, which is in agreement with data on its levels in circulation. Similar to the finding obtained in SAT, fetuin-A mRNA could not be detected by RT-PCR from PBMCs, in contrast to the case in HepG2 cells (Fig. 2b).

Moreover, to compare the presence of fetuin-A in cell lines from humans and rodents, immunodetection was performed in macrophages, preadipocytes and differentiated 3T3-L1, along with human plasma and serum as positive controls. As displayed in Fig. 2c, fetuin-A was present in human (THP-1) and murine macrophages (RAW264.7) but was undetectable in 3T3-L1 cells. Interestingly, fetuin-A transcripts were only detected in HepG2 cells, whereas a weak band corresponding to a lower size was detected in murine 3T3-L1 and RAW264.7 cells but not in PBMCs and THP1 human cells (Fig. 2d).

**Fig. 3:**
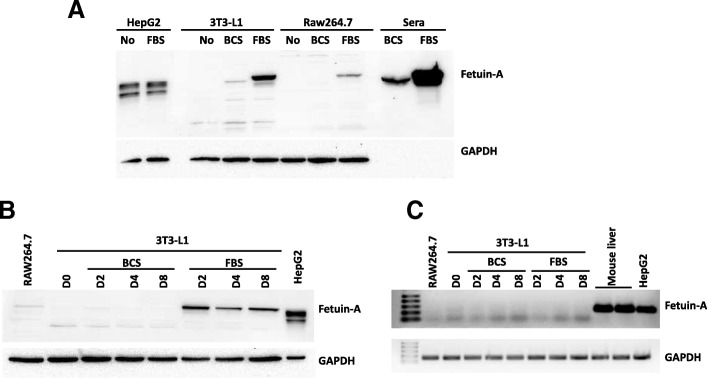
Effect of sera used in cell culture media on fetuin-A levels. **a** Total proteins were extracted from cell lines and subjected to Western blotting. The blots shown here are representative of independent experiments with consistent results. Proteins extracted from HepG2 cells were used as a positive control and loaded at a level 10 times lower than that in other cells. **b** Total proteins were extracted from 3T3-L1 cells at various stages of differentiation cultured in the presence of BCS or FBS. RAW264.7 and HepG2 were used as controls. HepG2 total proteins were loaded in the gel at a level 10 times lower than that in the other cells. **c** Total RNA was isolated and analysed using qRT-PCR from 3T3-L1 cells at various stages of differentiation cultured in the presence of BCS or FBS. RAW264.7, HepG2 and mouse liver biopsy were used as controls

Fetuin-A is commonly used as a supplement to cell culture media to enhance cell attachment and growth. Furthermore, some culture media are supplemented with BCS, whereas others are supplemented with FBS, depending on the cell line. This prompted us to assess the presence of fetuin-A in various cells cultured in media with and without FBS and BCS. Accordingly, higher fetuin-A content was observed when 3T3-L1 and RAW264.7 cells were cultured in the presence of FBS, whereas HepG2 fetuin-A levels were not affected upon culture with and without FBS, suggesting that a large proportion of fetuin-A is taken up from the culture medium by cell lines (Fig. 3a). To eliminate the possibility that fetuin-A expression in 3T3-L1 cells is linked to their maturity, these cells were differentiated into adipocytes in medium containing either BCS or FBS, which revealed that fetuin-A was present in cells only when FBS was used (Fig. 3b). The potential induction of fetuin-A expression in cells due to other factors present in FBS and not in BCS can be eliminated because the fetuin-A transcript was not detectable in those cells cultured in the presence of BCS and FBS, in contrast to the findings with HepG2 or mouse liver (Fig. 3c).

## Discussion

In the present study, the levels of fetuin-A in the plasma and SAT of diabetic and non-diabetic obese subjects and their modulation by physical exercise were assessed. Our main findings were: (i) fetuin-A levels in circulation were comparable between the two groups, whereas fetuin-A levels in SAT were increased significantly in the diabetic obese subjects; (ii) fetuin-A levels were mainly associated with diabetic markers (FB, HbA1c); (iii) the presence of fetuin-A in SAT and PBMCs was because of its uptake from circulation rather than its endogenous expression and (iv) 3 months of moderate physical exercise decreased fetuin-A levels in both circulation and SAT.

Fetuin-A was previously reported to be an independent risk factor for diabetes by positively associating with FBG and HbA1c [6, 31] and was elevated in the plasma of subjects with impaired glucose tolerance [32, 33] with or without NAFLD [34]. Nevertheless, other studies have reported lower fetuin-A levels in high-risk diabetes patients with vascular complications [35]. Similarly, no difference in fetuin-A levels was observed among obese elderly adults with or without metabolic syndrome [36]. In line with this, in our study, only a marginal increase in fetuin-A levels was observed in the diabetic obese, despite their significant differences in key insulin resistance and cardiometabolic parameters compared with their non-diabetic obese counterparts. These discrepancies in the reported data might be linked to the duration of diabetes and its related complications as well as the age of subjects in the study groups. Treatment regimen might also attenuate the expected increase of fetuin-A, as metformin and β-blockers have been reported to decrease its plasma levels [37, 38]. Furthermore, our study included only obese subjects, but other conditions such as fatty liver might also affect fetuin-A circulating levels linked to hyperglycaemia and diabetes; this is supported by a report describing that high fetuin-A levels are associated with fat accumulation in the liver [6].

Recent studies have investigated the modulation of fetuin-A by NAFLD in vascular complications such as CAD and PAD. Interestingly, fetuin-A levels were higher in CAD patients and associated with NAFLD but not in PAD patients suggesting that the crosstalk between fetuin-A, NAFLD and atherosclerosis varies according to the arterial site [17]. Furthermore, this interplay was also suggested to impair renal function through inflammatory signals by renal sinus fat cells around renal arteries in the presence of NAFLD [39]. Moreover, the same group also reported that fetuin-A is mediating crosstalk of fatty liver with islets inducing obesity-linked glucose blindness of beta cells [40].

Nevertheless, a recent study has reported a strong association of fetuin-A with the risk of diabetes that could not be explained by liver fat [41]. In contrast, glucose and hyperglycaemia were reported to increase the fetuin-A gene promoter activity and its expression in the liver [42]. The clear association of fetuin-A with features of diabetes, but not with obesity markers as observed in our study, further supports this latter report. This is reflected by our further comparison of fetuin-A levels in SAT from lean subjects with and without diabetes, which revealed increased fetuin-A protein levels in diabetic SAT (Additional file 6: Figure S3). Similarly, in obese subjects, SAT of diabetic subjects displayed higher levels of fetuin-A staining than that of non-diabetic subjects. In this context, resistance to the development of obesity was revealed in mice with fetuin-A knockout [4]. In contrast, fetuin-A gene variation was found to be not associated with body fat distribution [43], and recently, adipose tissue density was reported to be associated with various adipokines, except fetuin-A [44]. Furthermore, and despite the mechanistical evidence linking fetuin-A and insulin sensitivity, causal link of circulating fetuin-A with diabetes was not supported by a recent large Mendelian study in the general population [10].

Upon dividing our diabetic group in those with controlled or uncontrolled HbA1c levels, fetuin-A levels did not significantly change between these two groups, in line with a previous study that evaluated the effects of acute and long-term glucose control on this variable [45]. Interestingly, when focused on non-diabetic obese subjects separated into MHO and MUO groups, a significantly higher fetuin-A level was observed in MUO, as previously reported [46]. Accordingly, decreased circulating fetuin-A levels in the MHO group could be due to lower liver fat content, as previously reported when healthy and unhealthy subjects were compared [47]. In this context and despite unclear mechanisms, it was reported that fetuin-A inhibits the insulin receptor, which results in insulin resistance in adipocytes and skeletal muscle in rodents [5]. Further studies are needed to explain the crosstalk between fetuin-A and insulin resistance. Notably, a causal association between plasma levels of fetuin-A and myocardial infarction risk was reported in the EPIC-Potsdam study [48]. In line with the potential involvement of fetuin-A in cardiovascular diseases, our results revealed its negative correlation with HDL, as was reported previously [33, 49].

The adoption of a healthy lifestyle, including physical exercise, is an important non-pharmacologic approach for preventing diabetes and its complications. From this perspective, the effect of moderate exercise intervention for 3 months was investigated on fetuin-A levels in our study population. Interestingly, our results showed that fetuin-A levels were significantly decreased in both study groups in circulation and SAT following the exercise intervention, aside from a tendency for a decrease in the glycaemic index. Notably, in the non-diabetic obese group, the beneficial effect of exercise was mainly reflected in decreases in insulin and obesity indexes, whereas in the diabetic group, it was reflected in a significant reduction in HbA1c levels. These trends reported here specifically in obese subjects agree with findings of previous studies, in which exercise improved HbA1c levels in diabetic adults [50] and reduced hyperinsulinaemia in a population at high risk for metabolic dysregulation [51].

Conflicting findings were described in previous reports on the effect of physical exercise on fetuin-A levels: exercise was reported to reduce, increase or have no effect on fetuin-A levels, depending on the study [52]. Accordingly, beneficial effects of long-term exercise (12 weeks) on insulin sensitivity were reported and related to decreased fetuin-A and FFAs, which resulted in less TLR4 signalling [53]. A previous study has reported a significant correlation between fetuin-A and insulin resistance in liver, but not in skeletal muscle, after exercise and suggested that the exercise-induced decrease of fetuin-A is mainly linked to hepatic glucose production, regardless the changes in systemic inflammation [54, 55]. However, our present data showed decreased circulating fetuin-A levels in both diabetic and non-diabetic subjects after exercise. Moreover, our observation that physical exercise overall seems to be more beneficial for non-diabetic obese further supports our previous data that it provides a better improvement of heat shock response and decreased inflammation in non-diabetic obese subjects [29] and provides extra molecular evidence on the importance of regular physical exercise in preventing diabetes.

**Fig. 4:**
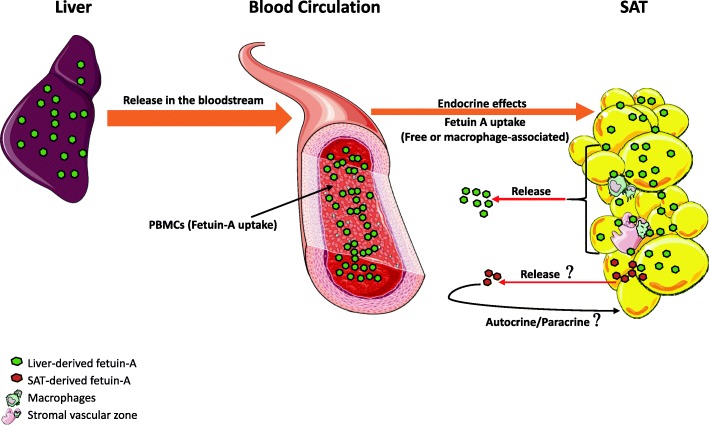
Proposed model for the presence of fetuin-A in white adipose tissue. Liver-derived fetuin-A is abundantly secreted into the bloodstream and acts as a hormone to promote metabolic effects on the targeted tissue, such as white adipose tissue (WAT). Fetuin-A from circulation is taken up by the WAT either directly and concomitantly with FFA or through macrophages infiltrating the WAT during chronic inflammation. The potential production and secretion of marginal levels of SAT-derived fetuin-A and their contribution to overall circulating fetuin-A levels are difficult to determine; however, if these occur, they probably act at the autocrine level in the interstitial compartment. Images were adapted from “Servier Medical Art”

Whether fetuin-A is produced by other tissues besides liver in adulthood remains unclear. Indeed, some reports have claimed that fetuin-A is only captured by these tissues from the circulating pool, in case of bones [26, 56], vascular wall cells [57] and kidney [27]. With regard to adipose tissue, so far only two studies have reported fetuin-A expression in SAT and VAT or its secretion by cultured adipocytes [24, 25]. Our results clearly showed an abundance of fetuin-A protein in SAT of our subjects, but we were unable to detect fetuin-A transcript in SAT. Other groups have also reported the absence of fetuin-A mRNA in human adipose tissue [58, 59]. Notably, we could abundantly detect adiponectin in SAT at both protein (Additional file 5: Figure S2) and mRNA levels (data not shown). Furthermore, fetuin-A protein in SAT was clearly increased in diabetic obese patients, but no mRNA was detected. In support with this finding, we further compared the levels of fetuin-A in SAT obtained from diabetic and non-diabetic lean subjects using samples from our previous study [29]. Our results showed that the levels of fetuin-A protein were increased with diabetes along with decreased adiponectin levels in lean subjects, as observed in obese subjects (Additional file 7: Figure S3). Overall, our data suggest that the majority, if not all, fetuin-A detected in SAT is taken up from the circulation. Consequently, adipose tissue potentially acts as a sponge for fetuin-A, similar to its function in the storage of FFAs, and offers temporary protection against excessive levels in circulation. Following physical exercise and/or in association with other physiological needs, fetuin-A is released from SAT back to circulation, as suggested in our model (Fig. 4). However, further investigation of this hypothesis is required because in this study, plasma fetuin-A was also decreased after exercise.

In support of the suggested process of uptake of fetuin-A, our results clearly showed the presence of fetuin-A protein in human PBMCs but not the presence of its transcript. Using various cell lines and culture conditions, our results further confirmed the uptake of fetuin-A from the surrounding medium in the case of adipocytes. Indeed, 3T3-L1 preadipocytes and differentiated adipocytes cultured in medium containing BCS, known to be poor in fetuin-A, did not display the presence of fetuin-A, whereas it was abundant in these cells when cultured in the presence of FBS, known to be rich in fetuin-A. Similarly, human and murine macrophage cell lines THP-1 and RAW264.7 cultured in their standard media supplemented with FBS showed clear increases in fetuin-A content. In contrast, fetuin-A levels in HepG2 cells did not change between these two culture conditions. These interesting observations are clearly related to the known high fetuin-A levels in FBS, but not in BCS and thus, its uptake from the culture medium by these cells. The hypothesis that specific factors present in FBS and not BCS induce fetuin-A expression in these cells can be eliminated because we were unable to detect fetuin-A transcript even under conditions of culture in FBS. A key observation in SAT of our subjects, in support of the occurrence of uptake of fetuin-A, was the presence of fetuin-A in vesicular structures, which was probably due to the infiltration of macrophages that were reported to store fetuin-A in such structures [30, 60]. Finally, the presence of different fetuin-A bands in the Western blot, depending on used cell lines and sera, is probably linked to various forms of fetuin-A due to its post translational modification as previously reported [61, 62].

The present study has some limitations. First, we did not have access to visceral adipose tissue biopsies, which would be more relevant to the pathophysiology of diabetes and obesity. Second, dietary intake was not controlled in our study, which may have affected fetuin-A levels and efficacy of physical exercise. Third, we did not directly measure insulin sensitivity using the gold-standard euglycaemic clamp. Forth, we did not have access to liver biopsies from our subjects. Indeed, if available, this might shed the light on the liver status with regards to NAFLD, however, this type of biopsies is neither ethically justifiable nor applicable in large studies. With regards to the study design, despite our adjustment for some confounders (age, gender, BMI), we cannot exclude the possibility that some of the associations we have observed can be explained by unmeasured or residual confounding. Furthermore, diabetes is associated with aging and thus it was challenging to find age-matched healthy obese. Finally, treatment regimen such as metformin and angiotensin receptors blockers have lowering effects on circulating fetuin-A levels [37, 38], therefore, possible impact of therapy in our study cohort may not be ruled out. However, this study has the strength that it involved analysis of a high-risk group of obese and diabetic adults who were undergoing a supervised moderate exercise protocol as an attractive behavioral approach to improve global health without drastic diet restriction.

## Conclusion

Fetuin-A was directly linked to markers of insulin resistance as well as diabetic risk in obese population. Physical exercise might ameliorate metabolic homeostasis by reducing fetuin-A levels. Our data also indicated that fetuin-A mainly acts as a hepatokine that promotes metabolic dysfunction in obesity and diabetes. However, further study is needed to unravel the mechanism of interaction between elevated fetuin-A level and insulin resistance and to delineate whether the presence of fetuin-A in adipose tissues and PBMCs is associated with its local production and/or simple storage.

## Additional files

Additional file 1: **Table S1.** Physical, biochemical and clinical characteristics of the diabetic group based on HbA1c levels. (DOCX 16 kb)

Additional file 2: **Figure S1.** Correlation analysis. Correlation of circulating fetuin-A with HbA1c, TG, HDL and FBG were assessed in the whole population using Spearman’s rank correlation coefficient. (PPTX 161 kb)

Additional file 3: **Table S2.** Physical, clinical and biochemical characteristics of the non-diabetic group based on MHO and MUO classification. (DOCX 18 kb)

Additional file 4: **Table S3.** Effect of exercise and diabetes and their combination on the characteristics of the study population. (DOCX 23 kb)

Additional file 5: **Figure S2.** Adiponectin levels and effect of exercise on the subcutaneous adipose tissue (SAT) of obese subjects with and without diabetes. Immunofluorescence analysis of adiponectin expression in SAT from obese subjects with and without diabetes before and after a 3-month physical exercise intervention (*n* = 10 for each group). Data were quantified as detailed in the Materials and Methods section. The *p* value was determined using Mann–Whitney test for comparisons between the diabetes and non-diabetes groups and using a paired *t*-test for intragroup comparisons before and after exercise. * *p* < 0.05 between diabetes and non-diabetes groups and # *p* < 0.05 between before and after exercise. (PPTX 145 kb)

Additional file 6: **Table S4.** Primer sequences used for quantitative real time PCR to analyze fetuin-A and GAPDH gene expression status. (DOCX 13 kb)

Additional file 7: **Figure S3.** Immunofluorescent analysis of fetuin-A levels in the subcutaneous adipose tissue (SAT) of lean subjects with and without diabetes. (A) Confocal microscopy showing fetuin-A abundance and localisation in vesicular-like structures in the thin rim of the cytoplasm of SAT collected from lean subjects with and without diabetes. (B) Representative confocal immunofluorescence images illustrating fetuin-A and adiponectin abundance in SAT from lean subjects with and without diabetes (*n* = 3 for each group). (PPTX 224 kb)

## Abbreviations

BCS: bovine calf serum
DBP: diastolic blood pressure
FRC: Fitness and Rehabilitation Center
HbA1c: haemoglobin A1c
HR: heart rate
IGT: impaired glucose tolerance
MHO: metabolically healthy obese
MUO: metabolically unhealthy obese
NAFLD: non-alcoholic fatty liver disease
NCEP: National Cholesterol Education Program
PBF: percent body fat
PBMC: peripheral blood mononuclear cell
SAT: subcutaneous adipose tissue
SBP: systolic blood pressure
VO_2,max_: maximum oxygen consumption
WAT: white adipose tissue
WC: waist circumference
