# Supplementary material for: Fetuin-A levels are increased in the adipose tissue of diabetic obese humans but not in circulation
Source: Lipids Health Dis. 2018 Dec 22;17:291. doi: 10.1186/s12944-018-0919-x (PMC6303986; doi:10.1186/s12944-018-0919-x)
Supplement: Supplementary file 1 — Table S1. Physical, biochemical and clinical characteristics of the diabetic group based on HbA1c levels. (DOCX 16 kb) [file 12944_2018_919_MOESM1_ESM.docx]

**Table S1** Physical, biochemical and clinical characteristics of the diabetic group based on HbA1c levels.

| Obese diabetic group | | | |
| --- | --- | --- | --- |
|  | HbA1c < 7 Subjects with good meta­bolic control | HbA1c ≥ 7 Subjects with poor meta­bolic control | P value |
| *Anthropometric and physical characteristics* | | | |
| Gender (Male/Female) | 44(25/19) | 72(38/34) | 0.671 |
| Age (years) | 53±7.7 | 51±9.4 | 0.257 |
| BMI (kg/m^2^) | 30.95 ±4.47 | 32.93 ±3.58 | **0.015** |
| PBF (%) | 36.48 ±12.26 | 36.95 ±5.07 | 0.824 |
| Waist (cm) | 101.75 ±10.75 | 108.31 ±9.44 | **0.004** |
| Hip (cm) | 110.80 ±11.39 | 111.70 ±12.40 | 0.717 |
| WBC10 | 7.10 ±2.15 | 7.53 ±1.84 | 0.278 |
| SBP (mmHg) | 115.00 ±11.09 | 122.14 ±10.39 | **0.002** |
| DBP (mmHg) | 74.21 ±6.83 | 76.96 ±5.70 | **0.044** |
| HR | 80.26 ±14.36 | 83.48 ±12.74 | 0.268 |
| V_O2, Max_ (ml/kg/min) | 16.26 ±4.91 | 15.44 ±3.94 | 0.397 |
| *Metabolic markers* | | | |
| Cholesterol (mmol/l) | 4.97 ±1.53 | 4.95 ±1.20 | 0.944 |
| HDL (mmol/l) | 1.25 ±0.61 | 1.14 ±0.33 | 0.296 |
| LDL (mmol/l) | 3.12 ±1.22 | 3.03 ±1.34 | 0.735 |
| TG (mmol/l) | 1.51 ±0.98 | 1.82 ±1.28 | 0.147 |
| FBG (mmol/l) | 6.49 ±1.92 | 9.48 ±3.37 | **<0.001** |
| HbA1c (%) | 6.08 ±0.55 | 8.95 ±1.48 | **<0.001** |
| Insulin (ng/ml) | 3.67 ±1.64 | 4.07 ±2.05 | 0.296 |
| C-pep (ng/ml) | 3.12 ±3.62 | 4.59 ±6.12 | 0.157 |
| hsCRP (𝜇g/ml) | 4.14 ±3.01 | 6.59 ±4.86 | **0.024** |
| Fetuin-A (mg/ml) | 1.13 ±0.35 | 1.29 ±0.33 | 0.065 |

*Data are presented as mean ± SD. Percent body fat (PBF), Body mass index (BMI), Systolic blood pressure (SBP), Diastolic blood pressure (DBP), Triglycerides (TG), High density lipoprotein (HDL), Low density lipoprotein (LDL), C-peptide (C-pep), High-sensitive C-Reactive Protein (hsCRP). Good and poor glycemic control subjects classification are based on HbA1c levels following the American Diabetes Association suggestions (HbA1c ≥7% (53 mmol/mol) as poor and HbA1c <7% as good metabolic control) [63]. Nonparametric Mann-Whitney test was used to determine significance of difference in means between the two groups.*

**Reference:**

63. Association Ad. Standards of Medical Care in Diabetes-2016: Summary of Revisions. Diabetes Care. 2016;39(Suppl 1):S1–112.
