# Supplementary figures and images for: Fetuin-A levels are increased in the adipose tissue of diabetic obese humans but not in circulation
Source: Lipids Health Dis. 2018 Dec 22;17:291. doi: 10.1186/s12944-018-0919-x (PMC6303986; doi:10.1186/s12944-018-0919-x)

## Slide 1
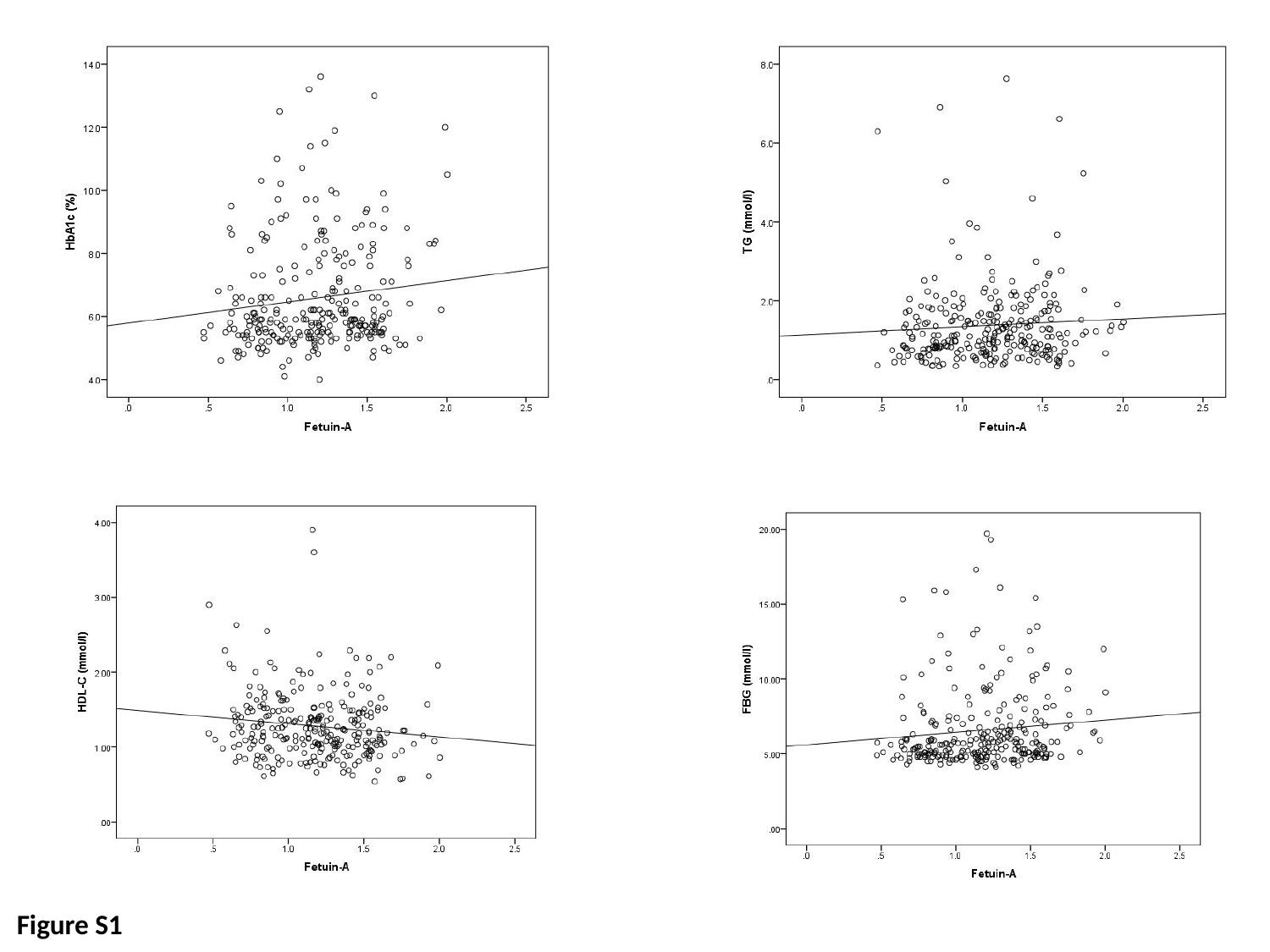

Figure S1

Supplement: Supplementary file 2 — Figure S1. Correlation analysis. Correlation of circulating fetuin-A with HbA1c, TG, HDL and FBG were assessed in the whole population using Spearman’s rank correlation coefficient. (PPTX 161 kb) [file 12944_2018_919_MOESM2_ESM.pptx]
