# Supplementary material for: Fetuin-A levels are increased in the adipose tissue of diabetic obese humans but not in circulation
Source: Lipids Health Dis. 2018 Dec 22;17:291. doi: 10.1186/s12944-018-0919-x (PMC6303986; doi:10.1186/s12944-018-0919-x)
Supplement: Supplementary file 3 — Table S2. Physical, clinical and biochemical characteristics of the non-diabetic group based on MHO and MUO classification. (DOCX 18 kb) [file 12944_2018_919_MOESM3_ESM.docx]

**Table S2** Physical, clinical and biochemical characteristics of the non-diabetic group based on MHO and MUO classification.

| Obese non-diabetic group | | | |
| --- | --- | --- | --- |
|  | MHO | MUO | P value |
| *Anthropometric and physical characteristics* | | | |
| Gender (Male/Female) | 64(24/40) | 97(39/59) | 0.730 |
| Age (years) | 39±11.1 | 43±12.7 | **0.017** |
| BMI (kg/m^2^) | 27.19 ±5.40 | 31.31 ±5.62 | **<0.001** |
| PBF (%) | 33.35 ±6.00 | 36.64 ±6.55 | **0.008** |
| Waist (cm) | 89.15 ±13.76 | 101.40 ±13.71 | **<0.001** |
| Hip (cm) | 105.57 ±9.01 | 114.36 ±13.70 | **<0.001** |
| WBC10 | 5.89 ±1.47 | 6.67 ±1.78 | **0.003** |
| SBP (mmHg) | 109.35 ±7.42 | 118.07 ±11.99 | **<0.001** |
| DBP (mmHg) | 70.87 ±5.09 | 76.28 ±7.30 | **<0.001** |
| HR (beats/min) | 81.54 ±10.43 | 78.32 ±10.26 | 0.107 |
| V_O2, Max_ (ml/kg/min) | 19.77 ±4.83 | 18.18 ±4.15 | 0.070 |
| *Metabolic markers* | | | |
| Cholesterol (mmol/l) | 5.03 ±0.96 | 5.13 ±0.92 | 0.545 |
| HDL (mmol/l) | 1.56 ±0.34 | 1.22 ±0.43 | **<0.001** |
| LDL (mmol/l) | 3.11 ±0.83 | 3.26 ±0.91 | 0.296 |
| TG (mmol/l) | 0.74 ±0.30 | 1.39 ±0.94 | **<0.001** |
| FBG (mmol/l) | 4.92 ±0.31 | 5.52 ±0.94 | **<0.001** |
| HbA1c (%) | 5.37 ±0.42 | 5.80 ±0.89 | **<0.001** |
| Insulin (ng/ml) | 3.01±1.39 | 3.70 ±2.34 | **0.042** |
| C-pep (ng/ml) | 4.66 ±5.03 | 5.72 ±7.14 | 0.343 |
| hsCRP (𝜇g/ml) | 4.33 ±5.39 | 5.05 ±3.42 | 0.588 |
| Fetuin-A (mg/ml) | 1.07 ±0.31 | 1.19 ±0.35 | **0.026** |

*Data are presented as mean ± SD. Percent body fat (PBF), Body mass index (BMI), Systolic blood pressure (SBP), Diastolic blood pressure (DBP), Triglycerides (TG), Low density lipoprotein (LDL), High density lipoprotein (HDL), C-peptide (C-pep), High-sensitive C-Reactive Protein (hsCRP). Non-diabetic subjects were segregated into two groups, metabolically healthy obese (MHO) and metabolically unhealthy obese (MUO) based on the Adult Treatment Panel-III (NCEP-ATPIII guideline) criteria for metabolic syndrome components [64] including the following criteria: (1) TG ≥150 mg/dL; (2) HDL-C <40 mg/dL for men and <50 mg/dL for women; (3) BP ≥130/85 mm Hg and (4) FPG≥100 mg/dL. Nonparametric Mann-Whitney test was used to determine significance of difference in means between the two groups.*

**Reference:**

64. National Cholesterol Education Program Expert Panel on Detection E. Treatment of High Blood Cholesterol in A: Third Report of the National Cholesterol Education Program (NCEP) Expert Panel on Detection, Evaluation, and Treatment of High Blood Cholesterol in Adults (Adult Treatment Panel III) final report. Circulation. 2002;106:3143–421.
