# Supplementary material for: Fetuin-A levels are increased in the adipose tissue of diabetic obese humans but not in circulation
Source: Lipids Health Dis. 2018 Dec 22;17:291. doi: 10.1186/s12944-018-0919-x (PMC6303986; doi:10.1186/s12944-018-0919-x)
Supplement: Supplementary file 4 — Table S3. Effect of exercise and diabetes and their combination on the characteristics of the study population. (DOCX 23 kb) [file 12944_2018_919_MOESM4_ESM.docx]

**Table S3** Effect of exercise and diabetes and their combination on the characteristics of the study population.

|  |  | DIABETES | EXERCISE | DIABETES | EXERCISE | DIABETES * EXERCISE |
| --- | --- | --- | --- | --- | --- | --- |
|  |  | Mean ± SD | Mean ± SD | Sig. | Sig. | Sig. |
| BMI (kg/m^2^) | Diabetic | 32.06 ±3.99 | 31.75 ±3.62 | **0.002** | 0.377 | **<0.0001** |
|  | Non-Diabetic | 29.00 ±4.99 | 28.34 ±4.58 |  |  |  |
| PBF (%) | Diabetic | 35.13 ±5.60 | 34.49 ±5.58 | 0.386 | 0.206 | 0.378 |
|  | Non-Diabetic | 33.98 ±5.99 | 33.04 ±6.32 |  |  |  |
| Waist (cm) | Diabetic | 107.26 ±9.60 | 105.47 ±10.02 | **<0.0001** | 0.193 | **<0.0001** |
|  | Non-Diabetic | 95.03 ±13.16 | 91.16 ±12.50 |  |  |  |
| Hip (cm) | Diabetic | 110.96 ±9.51 | 111.01 ±9.12 | 0.236 | 0.128 | 0.062 |
|  | Non-Diabetic | 108.74 ±15.53 | 105.38 ±9.28 |  |  |  |
| WBC10 | Diabetic | 7.70 ±1.86 | 7.50 ±1.89 | **<0.0001** | 0.534 | **<0.0001** |
|  | Non-Diabetic | 6.05 ±1.66 | 5.98 ±1.60 |  |  |  |
| SBP (mmHg) | Diabetic | 118.75 ±15.00 | 121.43 ±8.23 | 0.134 | 0.734 | **0.010** |
|  | Non-Diabetic | 114.52 ±10.27 | 112.68 ±7.58 |  |  |  |
| DBP (mmHg) | Diabetic | 75.62 ±6.30 | 75.75 ±5.38 | 0.413 | 0.851 | 0.483 |
|  | Non-Diabetic | 74.19 ±6.72 | 74.03 ±5.54 |  |  |  |
| HR (beats/min) | Diabetic | 83.00 ±13.27 | 79.56 ±12.23 | 0.520 | 0.091 | 0.257 |
|  | Non-Diabetic | 82.48 ±9.13 | 77.30 ±12.56 |  |  |  |
| V_O2, Max_ (ml/kg/min) | Diabetic | 18.37 ±3.96 | 19.08 ±5.45 | **0.001** | **0.004** | **<0.0001** |
|  | Non-Diabetic | 19.20 ±3.85 | 22.22 ±5.09 |  |  |  |
| Cholesterol (mmol/l) | Diabetic | 4.84 ±1.27 | 4.33 ±0.91 | **0.053** | 0.224 | **<0.0001** |
|  | Non-Diabetic | 5.24 ±0.91 | 5.18 ±0.98 |  |  |  |
| HDL (mmol/l) | Diabetic | 1.11 ±0.42 | 1.06 ±0.32 | **0.001** | 0.611 | **<0.0001** |
|  | Non-Diabetic | 1.44 ±0.52 | 1.40 ±0.43 |  |  |  |
| LDL (mmol/l) | Diabetic | 2.97 ±1.11 | 2.65 ±0.91 | 0.075 | 0.461 | **0.005** |
|  | Non-Diabetic | 3.28 ±0.90 | 3.23 ±0.90 |  |  |  |
| TG (mmol/l) | Diabetic | 1.66 ±1.01 | 1.60 ±0.73 | **<0.0001** | 0.283 | **0.001** |
|  | Non-Diabetic | 1.01 ±0.514 | 1.30 ±1.11 |  |  |  |
| FBG (mmol/l) | Diabetic | 8.09 ±2.96 | 8.17 ±2.98 | **<0.0001** | 0.534 | **<0.0001** |
|  | Non-Diabetic | 5.21 ±0.579 | 5.49 ±0.98 |  |  |  |
| HbA1c (%) | Diabetic | 7.75 ±1.96 | 7.10 ±1.35 | **<0.0001** | 0.087 | **<0.0001** |
|  | Non-Diabetic | 5.87 ±1.10 | 5.70 ±0.450 |  |  |  |
| Insulin (ng/ml) | Diabetic | 3.77 ±1.74 | 3.52 ±2.13 | 0.899 | 0.066 | 0.150 |
|  | Non-Diabetic | 3.92 ±2.11 | 3.05 ±1.36 |  |  |  |
| C-pep (ng/ml) | Diabetic | 3.53 ±5.02 | 3.37 ±3.36 | 0.084 | 0.559 | 0.085 |
|  | Non-Diabetic | 6.76 ±8.49 | 5.80 ±5.94 |  |  |  |
| hsCRP (𝜇g/ml) | Diabetic | 3.53 ±2.19 | 4.37 ±4.39 | 0.501 | 0.263 | 0.561 |
|  | Non-Diabetic | 4.68 ±6.60 | 3.21 ±1.95 |  |  |  |
| Fetuin-A (mg/ml) | Diabetic | 1.21 ±0.33 | 1.08 ±0.44 | 0.337 | **0.045** | 0.302 |
|  | Non-Diabetic | 1.15 ±0.31 | 1.07 ±0.45 |  |  |  |

*Data are presented as mean ± SD. Percent body fat (PBF), Body mass index (BMI), Systolic blood pressure (SBP), Diastolic blood pressure (DBP), Triglycerides (TG), High density lipoprotein (HDL), Low density lipoprotein (LDL), C-peptide (C-pep), High-sensitive C-Reactive Protein (hsCRP). Sig (significant) of 2-way ANOVA.*
