# Supplementary material for: Fetuin-A levels are increased in the adipose tissue of diabetic obese humans but not in circulation
Source: Lipids Health Dis. 2018 Dec 22;17:291. doi: 10.1186/s12944-018-0919-x (PMC6303986; doi:10.1186/s12944-018-0919-x)
Supplement: Supplementary file 5 — Figure S2. Adiponectin levels and effect of exercise on the subcutaneous adipose tissue (SAT) of obese subjects with and without diabetes. Immunofluorescence analysis of adiponectin expression in SAT from obese subjects with and without diabetes before and after a 3-month physical exercise intervention (n = 10 for each group). Data were quantified as detailed in the Materials and Methods section. The p value was determined using Mann–Whitney test for comparisons between the diabetes and non-diabetes groups and using a paired t-test for intragroup comparisons before and after exercise. * p < 0.05 between diabetes and non-diabetes groups and # p < 0.05 between before and after exercise. (PPTX 145 kb) [file 12944_2018_919_MOESM5_ESM.pptx]

## Slide 1
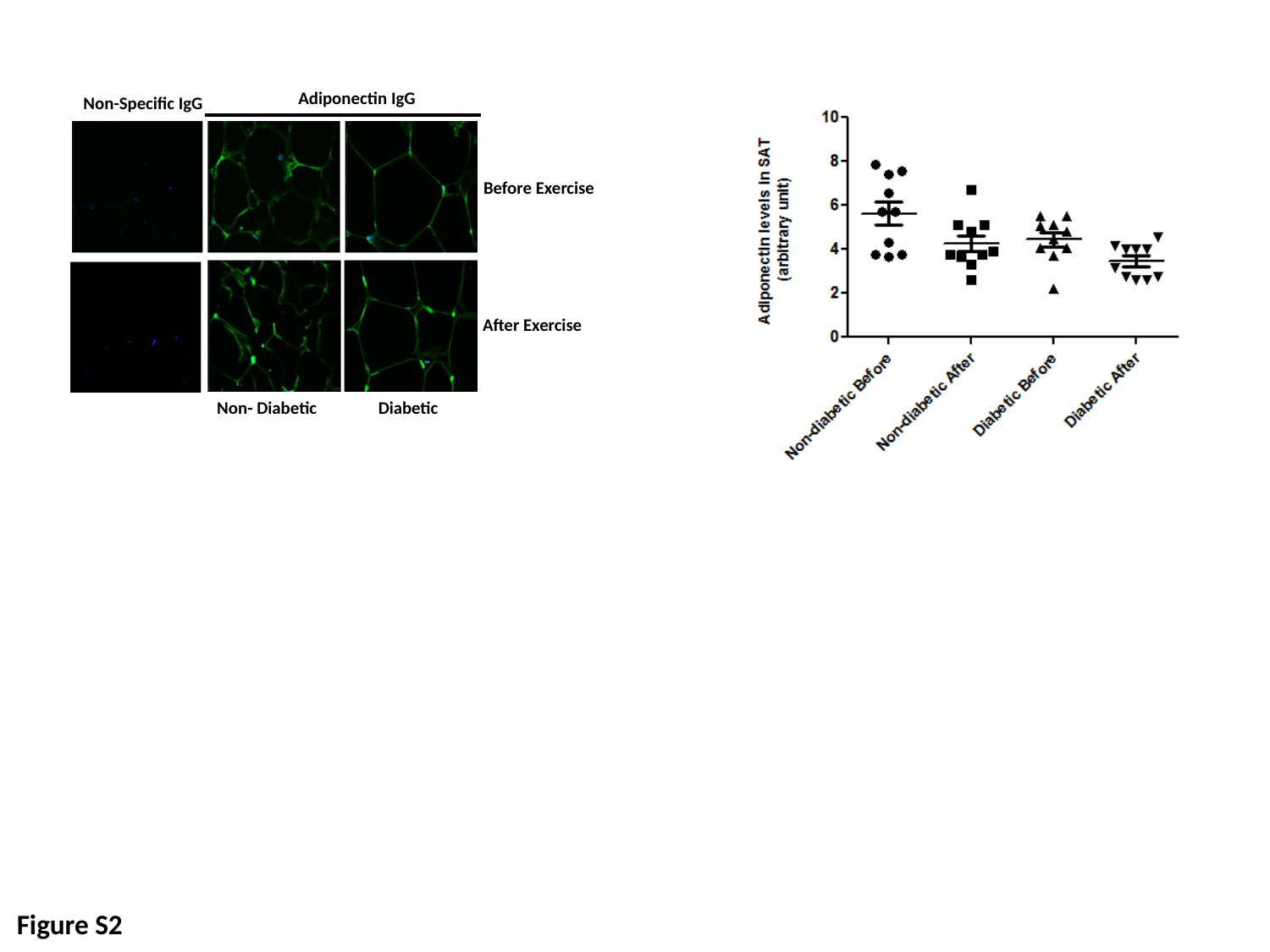

Adiponectin IgG
Non-Specific IgG
Before Exercise
After Exercise
Non- Diabetic
Diabetic
Figure S2
