# Supplementary material for: Fetuin-A levels are increased in the adipose tissue of diabetic obese humans but not in circulation
Source: Lipids Health Dis. 2018 Dec 22;17:291. doi: 10.1186/s12944-018-0919-x (PMC6303986; doi:10.1186/s12944-018-0919-x)
Supplement: Supplementary file 6 — Table S4. Primer sequences used for quantitative real time PCR to analyze fetuin-A and GAPDH gene expression status. (DOCX 13 kb) [file 12944_2018_919_MOESM6_ESM.docx]

**Table S4** Primer sequences used for quantitative real time PCR to analyze fetuin-A and GAPDH gene expression status.

| Genes | Forward primers | Reverse primers |
| --- | --- | --- |
| *Fetuin-A murine* | *5'-TTGCTCAGCTCTGGGGCT-3'* | *5'GGCAAGTGGTCTCCAGTGTGT-3'* |
| *Fetuin-A human* | *5'-CTTTGTCTTGCTCAGCTCTG-3'* | *5'-GGTGTCTATTTCAATCTCAAA-3'* |
