# Supplementary material for: Fetuin-A levels are increased in the adipose tissue of diabetic obese humans but not in circulation
Source: Lipids Health Dis. 2018 Dec 22;17:291. doi: 10.1186/s12944-018-0919-x (PMC6303986; doi:10.1186/s12944-018-0919-x)
Supplement: Supplementary file 7 — Figure S3. Immunofluorescent analysis of fetuin-A levels in the subcutaneous adipose tissue (SAT) of lean subjects with and without diabetes. (A) Confocal microscopy showing fetuin-A abundance and localisation in vesicular-like structures in the thin rim of the cytoplasm of SAT collected from lean subjects with and without diabetes. (B) Representative confocal immunofluorescence images illustrating fetuin-A and adiponectin abundance in SAT from lean subjects with and without diabetes (n = 3 for each group). (PPTX 224 kb) [file 12944_2018_919_MOESM7_ESM.pptx]

## Slide 1
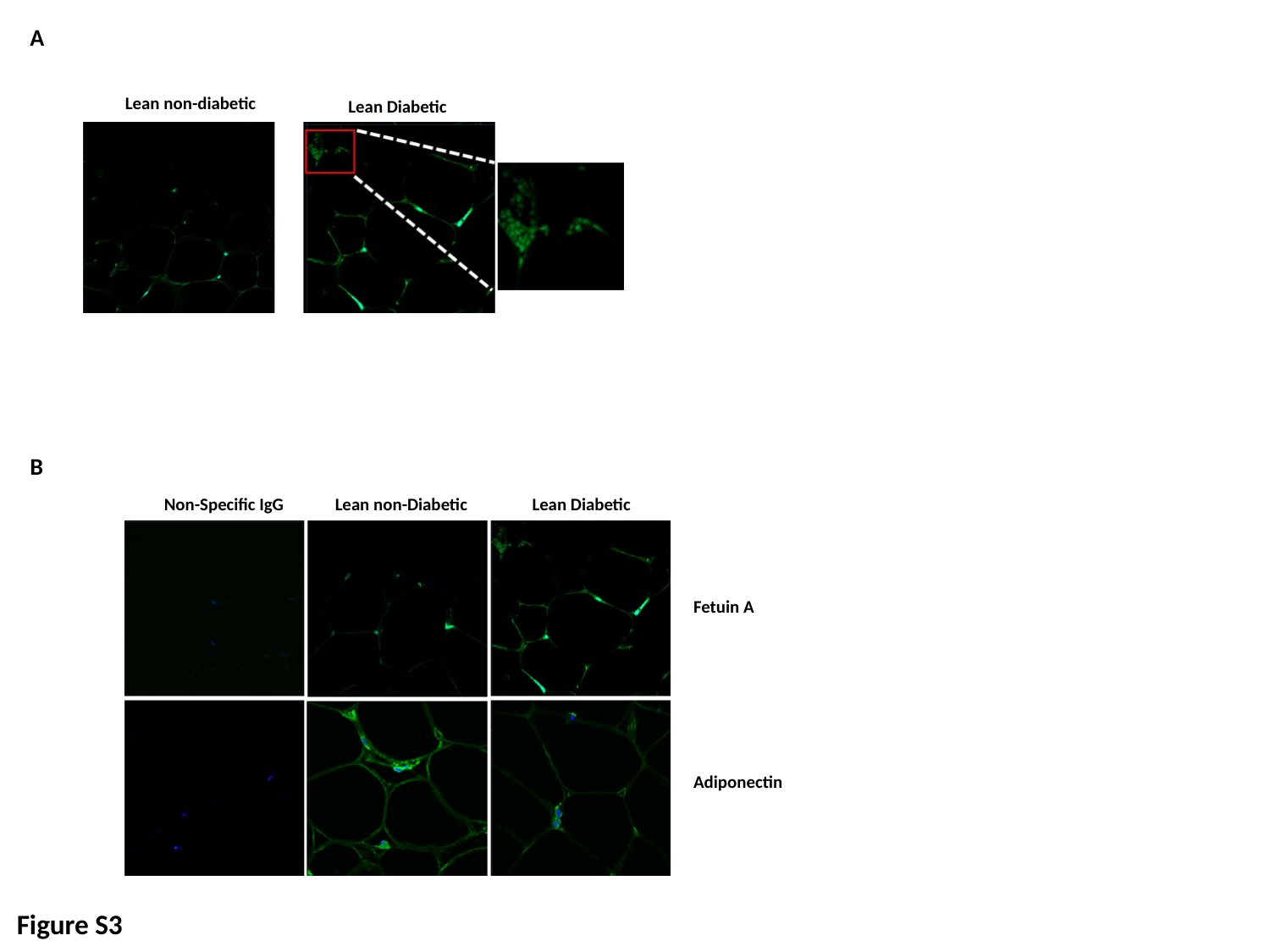

A
Lean non-diabetic
Lean Diabetic
B
Lean Diabetic
Lean non-Diabetic
Non-Specific IgG
Fetuin A
Adiponectin
Figure S3
